# Loss of Polycystin-1 Inhibits Bicc1 Expression during Mouse Development

**DOI:** 10.1371/journal.pone.0088816

**Published:** 2014-03-03

**Authors:** Peiwen Lian, Ao Li, Yuan Li, Haichao Liu, Dan Liang, Bo Hu, De Lin, Tang Jiang, Gilbert Moeckel, Dahui Qin, Guanqing Wu

**Affiliations:** 1 Division of Translational Cancer Research and Therapy, State Key Laboratory of Molecular Oncology, Cancer Hospital and Institute, Chinese Academy of Medical Sciences and Peking Union Medical College, Beijing, China; 2 Department of Medicine, Vanderbilt University, Nashville, Tennessee, United States of America; 3 Department of Cell & Developmental Biology, Vanderbilt University, Nashville, Tennessee, United States of America; 4 Department of Biochemistry, Vanderbilt University, Nashville, Tennessee, United States of America; 5 Department of Medicine, First Affiliated Hospital, Sun Yat-sen University, Guangzhou, China; 6 Department of Pathology, Yale University School of Medicine, New Haven, Connecticut, United States of America; 7 Department of Pathology, Moffitt Cancer Center, Tampa, Florida, United States of America; Children's National Medical Center, United States of America

## Abstract

*Bicc1* is a mouse homologue of *Drosophila Bicaudal-C* (*dBic-C*), which encodes an RNA-binding protein. Orthologs of *dBic-C* have been identified in many species, from *C. elegans* to *humans*. *Bicc1*-mutant mice exhibit a cystic phenotype in the kidney that is very similar to human polycystic kidney disease. Even though many studies have explored the gene characteristics and its functions in multiple species, the developmental profile of the *Bicc1* gene product (Bicc1) in mammal has not yet been completely characterized. To this end, we generated a polyclonal antibody against Bicc1 and examined its spatial and temporal expression patterns during mouse embryogenesis and organogenesis. Our results demonstrated that Bicc1 starts to be expressed in the neural tube as early as embryonic day (E) 8.5 and is widely expressed in epithelial derivatives including the gut and hepatic cells at E10.5, and the pulmonary bronchi at E11.5. In mouse kidney development, Bicc1 appears in the early ureteric bud and mesonephric tubules at E11.5 and is also expressed in the metanephros at the same stage. During postnatal kidney development, Bicc1 expression gradually expands from the cortical to the medullary and papillary regions, and it is highly expressed in the proximal tubules. In addition, we discovered that loss of the *Pkd1* gene product, polycystin-1 (PC1), whose mutation causes human autosomal dominant polycystic kidney disease (ADPKD), downregulates Bicc1 expression *in vitro* and *in vivo*. Our findings demonstrate that Bicc1 is developmentally regulated and reveal a new molecular link between *Bicc1* and *Pkd1*.

## Introduction

Studies of animal mutant models for polycystic kidney disease (PKD) and of human PKD patients have identified more than 20 genes whose mutations can induce PKD phenotypes [1], [2], [3]. The continued study of these cyst-associated genes and their products will help elucidate the disease mechanism of human inherited polycystic kidney diseases, such as autosomal dominant and recessive PKD (ADPKD and ARPKD).

*Bicc1* is a mouse homologue of *Drosophila Bicaudal-C* (*dBic-C*), the orthologs of which are much conserved in many species, from *C. elegans* to *humans* [4], [5], [6], [7], [8]. Loss of *dBic-C* in *Drosophila* disrupts the direction of anterior follicle cell migration and affects anterior-posterior patterning, so that the resulting embryos lack heads and exhibit duplicated posterior segments instead [9], [10]. The *Xenopus* homologue of *Bicaudal-C* (*xBic-C*) is one of the few molecules that, when microinjected ectopically, causes endoderm formation in the absence of mesoderm induction [11]. Knockdown of the zebrafish homologue of *Bicaudal-C* (*zBic-C*) induces cystic kidneys *in vivo* [12].

The gene product of *Bicc1* (Bicc1) contains several conserved N-terminal KH domains and a conserved C-terminal SAM domain [13]. The KH domains bind target mRNAs [4]. Recent studies indicated that the KH domains enable Bicc1 to recruit specific miRNA precursors and associate with Dicer, to guide these nascent miRNAs to anchor their target mRNAs. The SAM domain is nonessential for mRNA binding, but it is required for the transfer of Bicc1-targeted mRNAs to P-body-associated AGO proteins for silencing [14]. Therefore, the gene product Bicc1 is thought to be an RNA-binding molecule that functions to regulate diverse proteins at the post-transcriptional level.

To study the functional role of Bicc1, some chemically-induced or natively occurring *Bicc1*-mutant mouse models (*jcpk* and *bpk*) have been identified and several gene-targeting mouse models have been established [5], [6], [15], [16], [17]. Although *Bicc1* mutations in these models result from different mutant Bicc1 proteins, all the models exhibit cystic phenotypes in the kidney that are very similar to human polycystic kidney disease. These mouse models can provide insight into the functional roles of Bicc1 during mouse development. Even though gene expression of *Bicc1* during mouse development has been previously reported [5], [6], [9],[14], the developmental profiles of Bicc1 protein during mammalian development remain uncharacterized. Here we generated a polyclonal antibody against Bicc1 and used it to examine the distribution patterns of Bicc1 during mouse embryogenesis and organogenesis. In addition, we investigated the molecular relationship of Bicc1 to other human cystoproteins and discovered that loss of polycystin-1 (PC1), the gene product of *Pkd1*, whose mutation causes human ADPKD, reduced Bicc1 expression *in vitro* and *in vivo*. Our findings demonstrate that Bicc1 is developmentally regulated and indicate that its normal function may require normal PC1 expression.

## Materials and Methods

### Antibodies and reagents

A pGEX3 GST expression vector (Amersham) was used as the backbone for producing Bicc1 GST-fusion proteins. The cDNA of mouse Bicc1 (Q711-D858) was inserted in-frame into the vector. The mBicc1-fused plasmid was used to transform Rosetta host cells (Novagen) to produce the GST-fusion antigen. The antigen was subcutaneously injected into New Zealand white rabbits, at 0.5 mg per injection. The anti-mBicc1 serum (mBicc1p) was generated as reported in our previous studies [18]. All the antisera product and the preimmune serum (Pre-IM) were affinity-purified before performing experiments.

The following antibodies, staining materials, and reagents were used: anti-HA, and anti-Flag, anti-β-actin, and DAPI (Sigma-Aldrich Inc.); anti-Tamm-Horsfall glycoprotein antibody (THG) (Applied Biological Materials Inc.); fluorescein *lotus tetragonolobus* lectin (LTL) (Vector Laboratories); anti-Aquaporin-2 (AQP2) antibodies (Abcam Inc.); anti-GST antibody and pBABE-Puro retroviral vector (Cell Biolabs Inc.); pCMV-tag4 expression vector (Stratagene Inc.); pSico Lentiviral vector system (Addgene Inc.).

### Mouse strains

Our *Pkd2*, *Pkd1*, and *Pkhd1* mutant mice were described in detail previously [19], [20], [21]. All the mouse models used in this study were backcrossed (over 10 times) to the *C57BL/6* inbred background. The animal protocol was approved by Vanderbilt University Institutional Animal Care and Use Committee (Permit Number: M/12/143).

### Western blotting and quantitative PCR

Western blot analyses were performed using protocols similar to those described previously [18], [22]. Briefly, proteins from cultured cells or tissues were extracted in lysis buffer (0.5% NP-40, 5% Sodium deoxycholate, 50 µM NaCl, 10 µM Tris-HCl (pH 7.5), 1% BSA), homogenized and centrifuged. Protein samples were solubilized in protein loading buffer and denatured by boiling. The samples were electrophoresed in 10% SDS-PAGE gels. The membranes were incubated with 5% milk at room temperature for one hour and blotted with mBicc1p antibody at room temperature for 4 hours and then were incubated with peroxidase-conjugate secondary antibodies (Sigma) and detected with enhanced chemiluminescence (ECL) (Amersham).

Quantitative PCR was performed using the iCycler iQ Real-Time PCR Detection System with the iQ SYBR Green Supermix kit (Bio-Rad). The *Pkd1* primers were (forward) 5′-GCT AAG CTA CAC TTC TCC TTT ATC CG-3′ and (reverse) 5′-ACT TCT TGG CAT CTT TCA TCC CAC-3′. The *Pkd2* primers were (forward) 5′-GCG TGG TAC CCT CTT GGC AGT T-3′ and (reverse) 5′-CAC GAC AAT CAC AAC ATC C-3′. The *Pkhd1* primers were (forward) 5′-CAA ATG CCA CAG CCC AAC AG-3′ and (reverse) 5′-CAG AAT GGT TAG GGG TGG GA-3′.

### Histology, immunofluorescence (IF), immunohistochemistry (IHC), and confocal microscopy

The detailed procedures used for histology, IF, and IHC staining were published previously [18], [23]. For the microscopic analysis, a Zeiss Axioplan 2IE research microscope and a Zeiss Axiovert 200 inverted microscope system with 10×, 20×, and 40× objectives were used.

### Renal epithelial cell lines and their cultures

Mouse inner medullary collecting duct (IMCD) cells were cultured using the conditions described in the American Type Culture Collection manual. IMCD cells with *Bicc1*-silencing (IMCD^shC4C^) were reported in our previous study [24].

**Figure 1:**
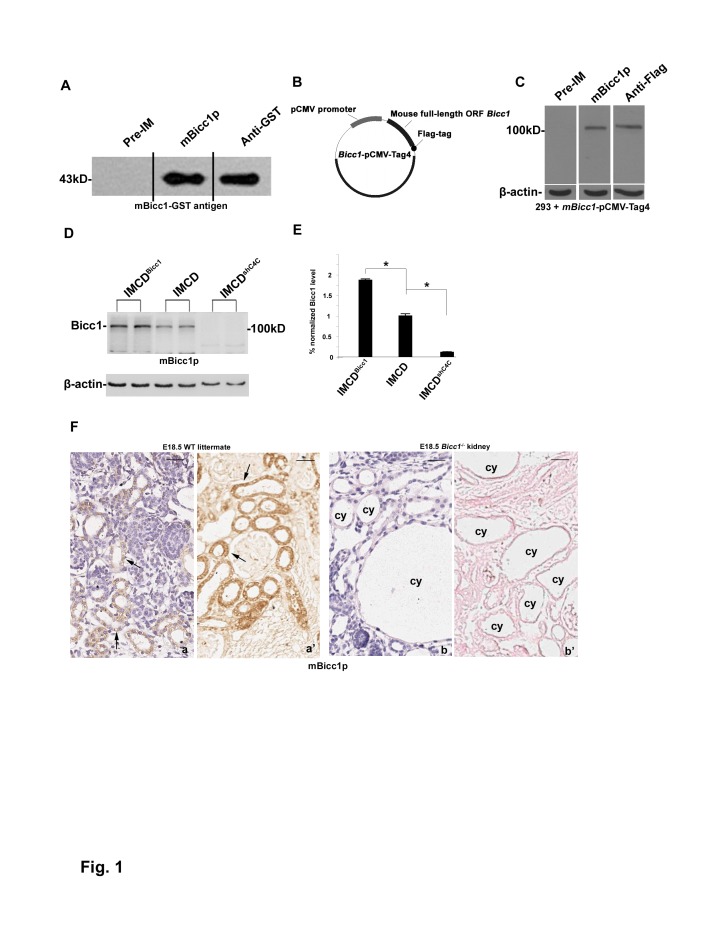
Specificity of the polyclonal antiserum mBicc1p for Bicc1. (A) The mBicc1-GST antigen was subjected to western blot analysis with the antibodies shown. The anti-Bicc1 (mBicc1p) and anti-GST antibodies, but not the preimmune serum (Pre-IM), recognized the mBicc1-GST antigen of the expected size (43 kD). (B) Schematic representation of the *Bicc1*-pCMV-Tag4 expression vector constructed by inserting the full-length ORF of *Bicc1* into Flag-tagged pCMV-Tag4. (C) *Bicc1*-pCMV-Tag4-transfected HEK293 cell lysates were subjected to western blot analysis with the antibodies shown. The anti-Bicc1 (mBicc1p) and anti-GST antibodies but not the Pre-IM recognized the *Bicc1*-pCMV-Tag4 protein of the expected size (∼110 kD). (D) Western analysis of duplicated protein lysates from wildtype, *Bicc1*-silenced (IMCD^shC4C^), and *Bicc1*-overexpressed IMCD cells (IMCD^Bicc1^) were blotted with the mBicc1p antibody. Compared to the wildtype control, the immunoreactivity was significantly increased in *Bicc1*-overexpressed IMCD cells and was almost not detected in the *Bicc1*-silenced cells. (E) Normalized quantitative analysis of the densitometry values of the western analyses. Compared to wildtype IMCD and *Bicc1*-silenced IMCD (IMCD^shC4C^) cells, *Bicc1*-overexpressed IMCD (IMCD^Bicc1^) cells showed significantly increased Bicc1 expression (*P<0.05). (F) Immunohistochemistry (IHC) staining of Bicc1 protein in the kidneys of E18.5 *Bicc1* ^−/−^ and its wildtype littermates. Positive staining (arrows) were showed in the wildtype kidney (a), while no obvious positive staining was seen in the E18.5 *Bicc1* ^−/−^ kidney (b). (a'–b') Corresponding areas of a–b were stained by Bicc1p IHC without counterstaining. Data presented are representative of two to three independently replicated experiments. “cy” = renal cysts. Bars: 25 µm in F.

To generate *Bicc1*-overexpressed stable cell line (IMCD^Bicc1^), we initially used Bicc1-pCMV-Tag4 clone (Fig. 1B) as backbone to insert mouse *Bicc1* ORF cDNA into LZRS-GFP vector (Addgene). Resulting LZRS-GFP-Bicc1-flag vector and pBABE-puro vector (Addgene) were co-transfected into HEK293 cells. At time points of cultured 48 and 72 hours, the viral-transfected supernatant was separately harvested and filtered with a 45 uM syringe filter. The 48 hour filtered supernatants were added on subconfluent cultured IMCD cells. After 24 hours, the infection was repeated with the 72 hour viral supernatants. One day later, puromycin was added for cell selection. Through a week Puromycin-selected culture, the remaining cells were resuspended and seeded on 100 mm culture plates (Costar) at a cell density of 1000 per plate. Once Puromycin-selected colonies formed, single colonies were picked and expend the colony into 24-well plates (Costar). A cloned cell line with *Bicc1*-overexpression, IMCD^Bicc1^, was identified by RT-PCR and confirmed by western blot analysis.

The null-*Pkd2* (E8) cell line, its maternally-derived *Pkd2* heterozygous (D3) cell line, the null-*Pkhd1* (M10H2) line, and its littermate-derived wildtype (W10B2) cell line were also described in our previous studies [23], [25].

The cells from null-*Pkd1* and its littermate-derived wildtype mice were generated by an approach similar to that reported in our previous studies [23], [25]. Briefly, the kidneys from E16.5 *Pkd1^−/−^* and wildtype littermate embryos with the *C57BL/6* congenic background were finely minced with a scalpel, and the minced tissue was incubated with 0.5% collagenase type IV at 37°C for 45 minutes and pipetted vigorously. The undigested tissue was removed by filtration through a 40 µm mesh filter. The remaining single cells and small organoids were washed three times with PBS containing 5 mM glucose. The cells were then incubated with 10 µg/ml biotinylated *Dolichos biflorus* agglutinin (DBA) (Vector, B-1035) at 4°C for 60 minutes. The cells were then washed again with PBS followed by incubation with 50 µl CELLectin Biotin binder Dynabeads (Dynal Biotech) at 4°C for 30 minutes. Since Dynabeads are superparamagnetic polystyrene beads, the incubated mixtures were then washed twice with PBS containing 5 mM glucose, using a magnetic rack. The cells were eluted with release buffer (Dynal Biotech) and were plated on 24-well dishes with LHC-9 Medium (Gibco) under 5% CO_2_ at 37°C overnight. The cells were then transferred to culture medium containing 5 units/ml murine IFN-γ (Peprotech Inc.), which was changed every other day, and placed in a 33°C incubator for at least 10 cell passages. The culture medium was then switched to 10% FCS DMEM/F12 (1∶1) (Gibco), and the cells were cultured for at least another 10 cell passages under the same culture conditions. Since the isolated collecting duct cells were not further cloned, the pool of cells (pool cells) was used for the current study.

*Pkd1* ^−/−^ pool cells with PC1-CT expression were produced by pSico Lentiviral vector system (Addgene) in which the entire human *PKD1* COOH-terminus was (V4102-R4278) constructed. Recombinant viruses were packaged and amplified on a large scale in HEK293 cells. Lentiviral particles were purified and infected *Pkd1* ^−/−^ pool cells following the introduction of the manufacturer.

Before being used in any cell-based assays, all the established cell lines were cultured under non-permissive conditions (37°C without IFN-γ) for at least three days to turn off the SV40 transgene activity.

### Statistics

All biochemical assays were repeated at least three times. Statistical analysis was performed where appropriate using the Student's t-test or one-way analysis of variance (ANOVA) followed by Tukey's Multiple Comparison Test. Differences with P-values <0.05 were considered statistically significant.

## Results

### Generation and characterization of anti-Bicc1 antiserum

A purified mouse Bicc1-GST fusion protein (mBicc1), encoding residues Q711 to D858 of Bicc1, was used to produce a rabbit polyclonal antiserum (mBicc1p) against the *Bicc1* gene product, Bicc1. The antiserum was generated and affinity-purified as described in our previous studies [18].

To test the specificity of mBicc1p, we performed western blot analyses of the antigen using the Pre-IM, mBicc1p, or an anti-GST antibody. No immunoreactive band was seen with Pre-IM, although strong positive bands of the expected size (∼43 kD) were detected with mBicc1p and the anti-GST antibody (Fig. 1A). The mBicc1p-positive band could be competed away with the mBicc1-GST antigen (data not shown), suggesting that the mBicc1p antiserum was specific for Bicc1.

To further confirm the specificity, we constructed a *Bicc1*-expression vector *Bicc1*-pCMV-Tag4, in which a Flag-tag was fused in-frame with the full-length ORF *Bicc1* cDNA (Fig. 1B). Western analyses showed that the transient transfection of HEK293 cells with *Bicc1*-pCMV-Tag4 yielded bands of the expected size (∼110 kD) that were immunoreactive with the mBicc1p and anti-Flag antibodies, but not with Pre-IM (Fig. 1C). We also used the mBicc1p antiserum to detect Bicc1 in lysates of IMCD cells, the stably *Bicc1*-overexpressed cell line IMCD^Bicc1^ and the *Bicc1*-silencing stable cell line IMCD^shC4C^ [24]. The ∼110-kD Bicc1 band was readily detected in wildtype IMCD cells and highly strong band was showed in the IMCD^Bicc1^ cells, whereas almost no band was seen in the IMCD^shC4C^ cells (Fig. 1D–E).

In addition, renal tissues from *Bicc1* knockout mice were also used for testing the specificity of mBicc1p antiserum. Strong immunohistochemistry (IHC) staining can be observed in E18.5 wildtype kidney, while there is no positive staining in its *Bicc1* ^−/−^ littermate (Fig. 1F). These results strongly indicated that the mBicc1p antiserum was specific for Bicc1.

### Bicc1 expression during mouse development

**Figure 2:**
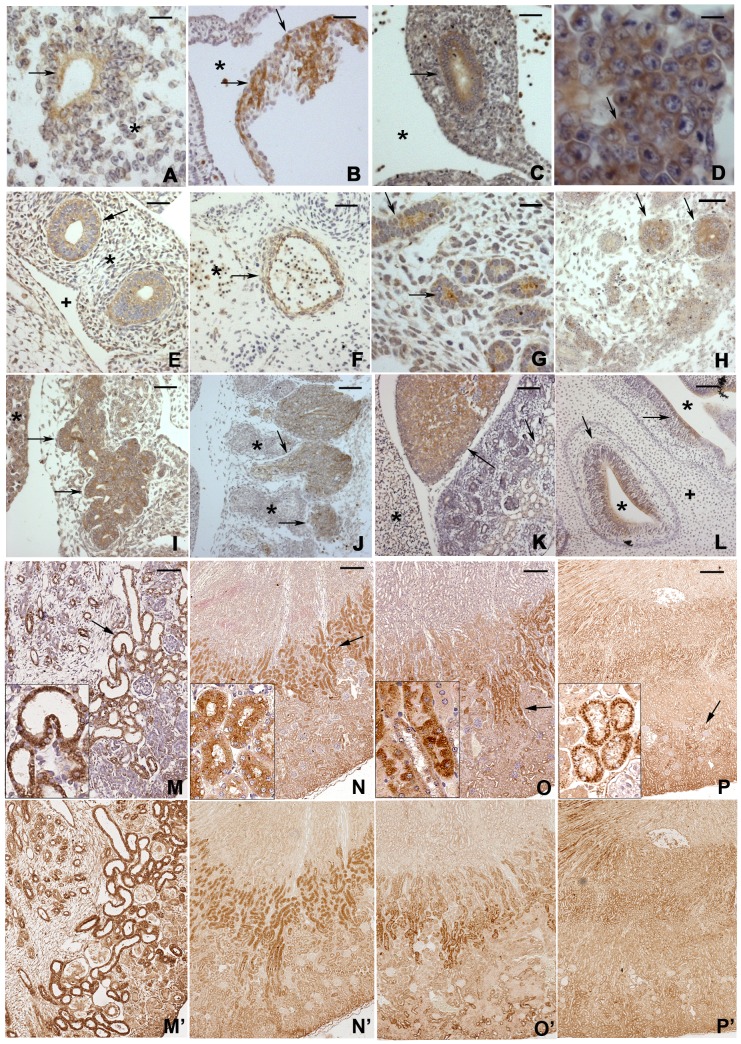
Expressional profiles of Bicc1 protein during embryogenesis and kidney development. By immunohistochemistry (IHC) staining (n≥3), (A) positive labeling is observed in the neural tube (arrow) at E8.5, * indicates the perineural tube mesenchymal tissue; (B) cardiomyocytes in the myocardial wall (arrows) at E9.5, * indicates the pericardio-peritoneal canal; (C) the primordial gut (arrow), * indicates the peritoneal cavity at E10.5; (D) immature hepatocytes (arrow) at E10.5; (E) epithelia of the main bronchi (arrows), + indicates the pericardio-peritoneal canal and * indicates the lung bud tissue; (F) the aortic wall (arrow), * indicates the cardinal vein; and (G) early ureteric bud (lower arrow)/mesonephric duct (upper arrow) at E11.5; (H) the renal comma-shaped body (arrows), (I) epithelia of the pancreatic primordium (arrows), * indicates hepatic tissue and (J) the posterior root ganglions at E12.5, * indicate vertebral bodies. Bicc1 appeared in (K) the adrenal cortex (lower arrow) and cortex of the kidney (upper arrow), * indicates the liver at E16.5; (L) olfactory epithelia (arrows) at E16.5, + indicates the primordial turbinate bone and * indicate the nasal cavities. (M) IHC with mBicc1p antiserum showed positive labeling (arrow) in the newborn mouse kidney. (N) In the 1-month-old kidney, positive staining (arrow) was observed in the juxta-medullary region of the kidney. (O) In the 3-month-old kidney, clear-cut Bicc1 expression (arrow) was seen a similar region of the kidney, but some positive labeling also appeared over the cortical region. (P) Besides of the juxta-medullary and cortical regions, positive labeling also extended to the medullary regions in the 6-month-old kidney. All high-power positive images were from the arrow-pointed regions and placed on the left-lower corners of M–O. (M'–P') showed IHC labeling without counterstaining for the corresponding regions of M–O, respectively. Bars: 10 µm in D; 20 µm in A, C, G–H; 25 µm in E and I; 50 µm in B, F, J–L; 100 µm in M; 150 µm in N–O; 300 µm in P.

To elucidate the patterns of Bicc1 protein expression during embryogenesis and primary organogenesis in mammals, mBicc1p was used for IHC analyses in developing mouse tissues. We found clear positive staining in the epithelial cells of the neural tube on embryonic day 8.5 (E8.5) (Fig. 2A), and then in the myocardial wall of the heart at E9.5 (Fig. 2B). By E10.5, the epithelial cells in the primordial gut and immature hepatocytes in the liver exhibited positive staining in their cytosol (Fig. 2C–D). By E11.5, Bicc1 expression was seen in the epithelia of the main bronchi, aorta, and early ureteric bud and mesonephric tubules, as the bud penetrated into the metanephrogenic mesenchyme (Fig. 2E–G). Clear positive staining continued to be observed in the renal comma-shaped body and the S-shaped body by E12.5 (Fig. 2H). At this stage, strong positive immunoreactivity could also be seen in the pancreatic primordium and posterior root ganglions (Fig. 2I–J). At E16.5, strong Bicc1 expression was observed in fasciculated cells in the developing adrenal cortex and in olfactory cells (Fig. 2K–L). By E16.5, the Bicc1 expression appeared to decrease in the cardiomyocytes and primordial gut (data not shown). The finding of Bicc1 protein profiling highly coincides with the studies by *Bicc1* mRNA in situ hybridization [5], [6], [9].

### Expression of Bicc1 in the postnatal mouse kidneys

Using the mBicc1p antibody, we next performed IHC analyses of the kidneys in newborn mice. Bicc1-positive staining was predominantly observed in the developing tubules of the kidney (Fig. 2M). By the age of 1 month, the Bicc1 expression was concentrated at the juxta-medullary region, with relatively weak expression in the cortical and medullary regions (Fig. 2N). At 3 months, although staining pattern was similar to that seen at 1 month, increased Bicc1 expression was seen over the cortical region of the kidney (Fig. 2O). At 6 months, the Bicc1 expression also extended into the medullary region (Fig. 2P). We also examined the kidneys of 9- and 12-month-old mice with the same antibody, but found no significant Bicc1 expression changes. The staining was mainly seen in the cytoplasm of the positive cells, suggesting that Bicc1 has a cytosolic distribution *in vivo* (Fig. 2M–P). Collectively, these results showed that Bicc1 expression is regulated during renal development.

### Bicc1 is expressed in multiple nephron segments of the adult mouse kidney

**Figure 3:**
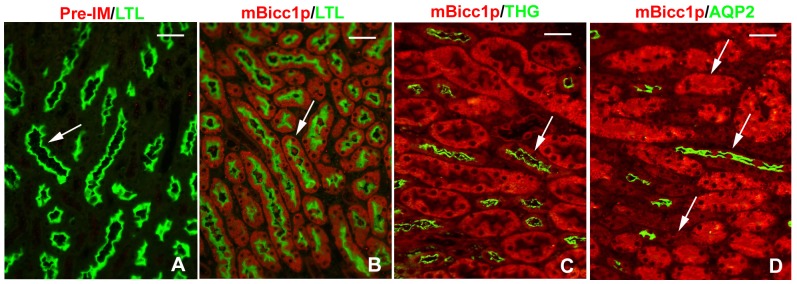
Bicc1 expression in renal nephrons. Double immunofluorescence staining with mBicc1p and nephronic markers (LTL, THG, and AQP2) in paraffin-embedded sections of adult wildtype mouse kidneys (n≥5). (A) The Pre-IM was used as a negative staining control. (B) Bicc1 (red) was co-expressed with LTL (green) in the epithelial cells of the renal proximal tubules; (C) Bicc1 (red) was also co-expressed with THG (green) in the epithelial cells of the Henle's thick ascending limbs; (D) With mBicc1p staining (red), Bicc1 was highly expressed in the renal proximal tubules (top arrow) and weakly expressed in the collecting ducts (lower arrow). mBicc1p co-staining with the anti-AQP2 antibody (green) showed weakly co-expression of Bicc1 and AQP2 in the epithelial cells of the collecting ducts (middle arrow). Bar: 30 µm in A–D.

The nephron segments with positive mBicc1p staining were identified by costaining with *Lotus tetragonolobus* lectin (LTL) for the proximal tubules (PT), Tamm-Horsfall glycoprotein (THG) for the medullary thick ascending limb of Henle (LH) and distal tubules (DT) [20], [26], and anti-Aquaporin-2 (AQP2) antibodies for the collecting ducts (CD). We examined the immunocytochemical distribution of Bicc1 in paraffin-embedded sections of wildtype adult kidneys. The Pre-IM from the same rabbit used to generate mBicc1p was used as the negative staining control (Fig. 3A). Under these conditions, Bicc1 showed diffuse cytoplasmic staining and was strongly co-expressed with the marker for epithelial cells of the proximal convoluted tubules (Fig. 3B), and distal tubules (Fig. 3C). Relatively weak-positive labeling was also seen in the collecting ducts of the adult kidneys (Fig. 3D). These results suggested that, although it is detected in all the nephronic segments, Bicc1 is highly expressed in the epithelial cells of the proximal convoluted tubules, which are derived from the metanephros, coincides with the previous report that this protein is co-expressed with proximal convoluted tubules of the kidney [14].

### Loss of *Pkd1* downregulates the Bicc1 expression *in vitro*

**Figure 4:**
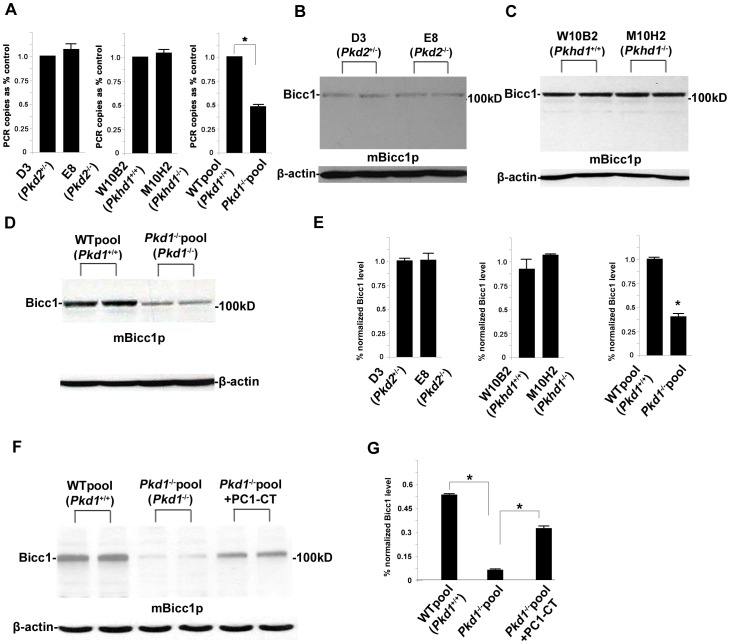
Loss of PC1 downregulates Bicc1 expression *in vitro*. (A) qPCR analysis of cultured cell lines with or without *Pkd1*, *Pkd2*, or *Pkhd1* showed no change in the *Pkd2* or *Pkhd1* mRNA expression, while *Bicc1* was significantly downregulated in the cells lacking *Pkd1* compared to the wildtype control (*P<0.05) (n = 3). (B) Western blot analysis of protein lysates from null-*Pkd2* cells (E8) and *Pkd2*-heterozygous cells (D3) using the mBicc1p antibody suggested that PC2 loss did not affect Bicc1 expression. (C) Similar western blots for null-*Pkhd1* cells (M10H2) and *Pkhd1*-wildtype cells (W10B2) showed that loss of *Pkhd1* also did not affect Bicc1 expression. (D) Western blot analysis for the null-*Pkd1* cells and their wildtype littermate cells showed that loss of *Pkd1* markedly downregulated the Bicc1 protein expression. (E) Normalized quantitative analysis of the densitometry values of the western analyses. Compared to null-*Pkd2* or -*Pkhd1* cells, only the null-*Pkd1* cells showed significantly reduced Bicc1 expression (*P<0.05). (F) With the wildtype littermate cell control, western blot analyses for the null-*Pkd1* pool cells and their PC1-CT transfected cells showed that the restoration of PC1 COOH-terminus markedly rescued the downregulation level of Bicc1 expression in the null-*Pkd1* cells. (G) Normalized quantitative analysis of the densitometry values of the western analyses. Compared to the null-*Pkd1* cells, the cells with PC1-CT transfection can partially restored Bicc1 expression in the null-*Pkd1* cells (*P<0.05). Data presented are representative of two to three independently replicated experiments.

To explore the molecular relationship between *Bicc1* and the other known human ADPKD and ARPKD causal genes, *Pkd1*, *Pkd2*, and *Pkhd1*, we first examined the *Bicc1* mRNA levels in *Pkd1* ^−/−^, *Pkd2* ^−/−^, or *Pkhd1* ^−/−^ cells using qPCR and compared them to the levels in their littermate-generated wildtype counterparts [23], [25]. Interestingly, only the cells lacking *Pkd1* exhibited a significant downregulation of *Bicc1* (Fig. 4A), suggesting that the loss of *Pkd1* inhibits *Bicc1* gene expression. To confirm this finding, we used the mBicc1p antibody in western blots of cell lysates from the littermate-derived, paired cell lines *Pkd2* ^+/−^(D3)/*Pkd2* ^−/−^(E8) [23], wildtype (W10B2)/*Pkhd1* ^−/−^(M10H2) [25], or wildtype/*Pkd1* ^−/−^. We found no differences in Bicc1 expression between the lysates of the *Pkd2* ^+/−^/*Pkd2* ^−/−^ and wildtype/*Pkhd1* ^−/−^ cells (Fig. 4B–C). By comparison, however, there was a marked decrease in Bicc1 expression in the lysates of the *Pkd1* ^−/−^ cells (Fig. 4D). The mRNA and western results together indicated that *Pkd1* ^−/−^ cells had approximately half the Bicc1 levels of their wildtype littermates (Fig. 4A, E), and suggested that both the mRNA and protein of *Bicc1* are downregulated by the lack of *Pkd1*.

To further validate this finding, we have restored the entire human PC1 COOH-terminus (PC1-CT) into *Pkd1* ^−/−^ cells by lentivirus system to determine if re-expression of PC1 can rescue the downregulation of Bicc1 in the null-*Pkd1* cells. The western blots of cell lysates from the littermate-derived cell lines: wildtype, and *Pkd1* ^−/−^, as well as *Pkd1* ^−/−^ cells transfected with the PC1-pSico vector (*Pkd1* ^−/−^+PC1-CT) showed that downregulation of Bicc1 of *Pkd1* ^−/−^ cells can be significantly rescued (Fig. 4F–G). This result strongly demonstrates that downregulation of Bicc1 expression in the *Pkd1* deficient cells is due to loss of PC1.

### Mice with null-*Pkd1* alleles also exhibit Bicc1 downregulation

**Figure 5:**
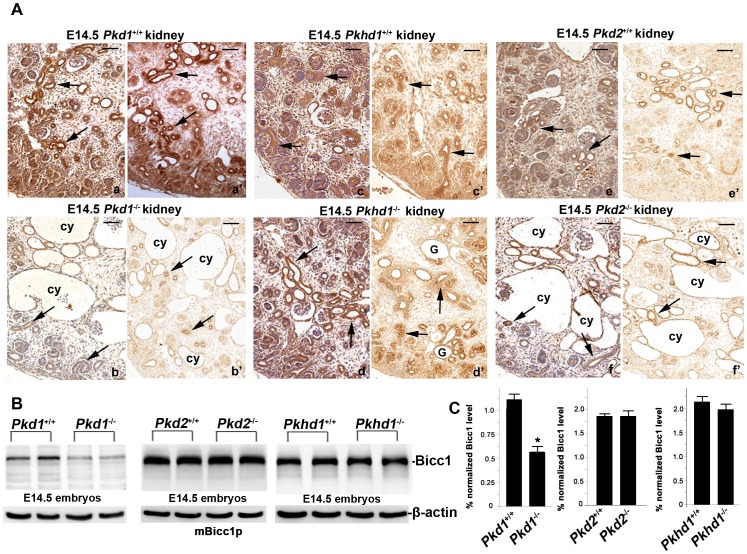
Loss of PC1 downregulates Bicc1 expression *in vivo*. (A) Positive mBicc1p IHC staining (arrows) in the mouse embryonic kidneys. The E14.5 wildtype (a) and its littermate *Pkd1* ^−/−^ (b) kidneys were stained by mBicc1p antibody (n≥3). In comparisons of kidney tissues with and without *Pkd1*, the null-*Pkd1* kidney showed significantly reduced Bicc1 expression. However, there are no Bicc1 expressional difference between the age-matched wildtype (c and e) and their littermate *Pkhd1* ^−/−^ (d) or littermate *Pkd2* ^−/−^ (f) kidneys, respectively. “cy” = renal cysts; “G” = glomerulus. (B) Compared to its wildtype littermate, western blot of duplicated tissue lysates from E14.5 embryos showed that loss of PC1 markedly downregulates Bicc1 protein level (left panel). A similar western blot showed equal immunoreactivities between the tissue lysates from E14.5 *Pkd2* ^−/−^ and its wildtype littermate (middle panel) and between the E14.5 *Pkhd1^−/−^* and its littermate wildtype embryos (n≥3). β-actin for protein loading control. (C) Normalized quantitative analysis of densitometry values of the western analyses. Compared tissues with and without *Pkd1*, *Pkd2* or *Pkhd1*, only null-*Pkd1* tissue shows significantly reduced Bicc1 expression (*P<0.05). Bars: 50 µm in A.

To validate this finding *in vivo*, we performed IHC with mBicc1p using embryonic kidneys from wildtype, *Pkd1*, *Pkd2*, or *Pkhd1* mice. Compared to wildtype, the Bicc1 expression was significantly decreased in the kidney of the *Pkd1* ^−/−^ littermates at E14.5 (Fig. 5Aa *vs.* b). However, the Bicc1 expression levels in E14.5 kidneys of *Pkhd1* ^−/−^ and *Pkd2* ^−/−^ mice seem similar to their wildtype littermates (Fig. 5Ac *vs.* d and Fig. 5Ae *vs.* f).

Finally, we have also performed western analyses from tissue lysates of E14.5 null-*Pkd2*, null-*Pkd1* and null-*Pkhd1* embryos, and their wildtype-littermates as well. Compared to wildtype, Bicc1 is also significantly downregulated in the null-*Pkd1* tissues. However, there are no any Bicc1 expression changes between the tissue lysates with and without *Pkd2* and *Pkhd1* (Fig. 5B–C). These *in vivo* results provide further evidence that the loss of *Pkd1* downregulates Bicc1 expression.

## Discussion

Studies using chemically-induced or natively occurring *Bicc1*-mutant mouse models (*jcpk* and *bpk*) and other *Bicc1*-gene-targeted mouse models have recently demonstrated that the disruption of *Bicc1* can induce polycystic kidney disease with phenotypes very similar to human ADPKD [5], [6], [17]. Although gene expression of *Bicc1* during mouse development has been previously reported [5], [6], [9], [14], the characteristics of *Bicc1* gene product, Bicc1, during embryogenesis and organogenesis in mammals has not been completely explored. In this study, we generated a new polyclonal antibody, mBicc1p, against the *Bicc1* gene product, Bicc1, and used it to study the expression of Bicc1 during mouse development. By using this antibody, we also discovered that the loss of *Pkd1* downregulates Bicc1 expression *in vitro* and *in vivo*, revealing a molecular relationship between *Bicc1* and *Pkd1*, a known causal gene for human ADPKD. Our findings demonstrate that Bicc1 expression is developmentally regulated and that its normal function may require PC1 expression.

To investigate the functional role of Bicc1 during mammalian development, several studies have examined mouse *Bicc1* gene expression patterns using *in situ* mRNA hybridization [6], [9]. By whole-mount *in situ* hybridization, *Bicc1* mRNA expression is first detected at the rostral tip of the primitive streak around E7.5 and in neural tissues at E8.5. At E13.5, strong *Bicc1* mRNA labeling is detected in the bone, heart, and lung tissues. In mouse kidney development, *Bicc1* mRNA is detected at the mesonephros and the first branch of the ureteric bud tree at E11.5, and then at the metanephros and the comma- and S-shaped bodies at E13.5. At birth, *Bicc1* mRNA is mainly seen in the proximal tubules of the mouse kidney. These *Bicc1* mRNA patterns and tissue distributions highly correspond to our findings with anti-Bicc1 antibody staining, further demonstrating the specificity of the mBicc1p antibody for Bicc1, and supporting protein expression profile described in this study.

Primary cilia are found on diverse cell types ranging from fibroblasts to epithelia [27]. These cilia are generally thought to function as extracellular sensors, for regulating cell behavior [28]. Many human and rodent cystoproteins, the mutants of which cause PKD phenotypes, have been reported to co-localize with the primary cilium/basal bodies of renal epithelia [1], [29]. However, previous reports indicate that Bicc1 was distributed to the P-bodies of renal epithelial cells [5], [6], [14]. This novel subcellular localization of Bicc1 indicated that the primary cilium/basal bodies in renal epithelial cells may not be the only targeted organelle for cystogenesis of the kidney.

We recently established stable IMCD (mouse inner medullary collecting duct) cell lines, in which *Bicc1* is silenced by short hairpin RNA (shRNA) expression. Our previous results showed that the inhibition of Bicc1 disrupts normal tubulomorphogenesis and induces the cystogenesis of IMCD cells grown in three-dimensional culture [24]. These cells also have a significant defect in E-cadherin-based cell-cell adhesion, along with abnormalities in actin cytoskeletal organization, cell-extracellular matrix interactions, ciliogenesis, and cell proliferation/apoptosis. Interestingly, these defects are also seen in epithelial cells lacking *Pkd1* and *Pkd2*, mutations that can cause human ADPKD [23], [30], [31]. We therefore assume that the cystogenesis resulting from the downregulation of *Bicc1* may be associated with a disruption in the normal *Pkd1* or *Pkd2* expression.

As predicted, a recent study demonstrated that the absence of *Bicc1* in cells promotes miR-17's binding to the 3′UTR of *Pkd2* and disrupts the stability of the *Pkd2* mRNA [6]. This finding indicates that *Bicc1* acts as a posttranscriptional factor upstream of *Pkd2* and reveals the molecular relationship between *Bicc1* and *Pkd2*, a causal gene of human ADPKD. In the present study, we showed that another human ADPKD causal gene, *Pkd1*, is involved in the regulation of *Bicc1* expression *in vitro* and *in vivo*. This finding indicates that *Bicc1* is not only associated with *Pkd2*, but also with *Pkd1* expression. The association between Bicc1 and polycystins implies that a disruption of *Bicc1* induces cystic phenotypes through the polycystin pathway.

In summary, we have newly generated a polyclonal antibody, mBicc1p, that is specific for the *Bicc1* gene product Bicc1. Using this antibody, we demonstrated the developmental profile of Bicc1 protein during embryogenesis and organogenesis in mammals. We found that the Bicc1 protein is expressed as early as E8.5 in the mouse neural tube and appears at the ureteric bud and nephronic tubules by E11.5. After birth, Bicc1 expression extends into the proximal tubules of the kidney, and thereby is predominately expressed in this nephron segment. Moreover, we discovered that normal Bicc1 expression requires PC1 expression. These findings together indicate that Bicc1 is a key protein for embryogenesis and organogenesis in mammals and uncover a new molecular link between *Bicc1* and *Pkd1*, whose mutation causes human ADPKD.
